# Eight new species of *Cestrum* (Solanaceae) from Mesoamerica

**DOI:** 10.3897/phytokeys.8.2238

**Published:** 2012-01-04

**Authors:** Alex K. Monro

**Affiliations:** 1 Department of Botany, The Natural History Museum, London SW2 5SY, UK

**Keywords:** Central America, Conservation Assessments, Costa Rica, Flora Mesoamericana, Guatemala, Mexico, Panama

## Abstract

As part of the preparation of a taxonomic revision of *Cestrum* (Solanaceae) for Flora Mesoamericana eight hitherto undescribed species from Mexico, Guatemala, Costa Rica and Panama were identified. These eight new species are described and illustrated. Affinities of the species are discussed and Global Species Conservation Assessments presented.The new species are *Cestrum amistadense* A.K. Monro, **sp. nov.** (Vulnerable) which most closely resembles *Cestrum longiflorum* Ruiz & Pav., *Cestrum contrerasianum* A.K. Monro, **sp. nov.** (Vulnerable) which most closely resembles *Cestrum formosum* C.V.Morton, *Cestrum darienense* A.K. Monro, **sp. nov.** (Near Threatened) which most closely resembles *Cestrum morae* Hunz., *Cestrum gilliae* A.K. Monro, **sp. nov.** (Near Threatened) which most closely resembles *Cestrum morae*, *Cestrum haberii* A.K. Monro, **sp. nov.** (Vulnerable) which most closely resembles *Cestrum poasanum* Donn.Sm., *Cestrum knappiae* A.K. Monro, **sp. nov.** (Near Threatened) which most closely resembles *Cestrum acuminatum* Francey, *Cestrum lentii* A.K. Monro, **sp. nov.** (Near Threatened) which most closely resembles *Cestrum johnniegentrianum* D’Arcy and *Cestrum talamancaense* A.K. Monro (Least Concern) which most closely resembles *Cestrum laxum* Benth.

## Introduction

The genus *Cestrum* (Solanaceae) includes ca. 150 species (Nee 1986, 2001; Benítez and D’Arcy 1998) of moth, butterfly and hummingbird pollinated small trees, shrubs, vines and robust herbs from the New World tropics and subtropics. Within the Solanaceae *Cestrum* is characterised by nearly actinomorphic flowers, small, persistent calyces, long-tubular corollas, small longitudinally dehiscent anthers held close to the corolla mouth, superior ovaries (Benítez and D’Arcy 1998) and few-seeded berries. *Cestrum* also frequently has truncated side branches subtended by a leaf at each node which can give the superficial appearance of an opposite arrangement of unequal leaves. Cytologically, *Cestrum*, together with its sister genus *Sessea* is unusual for the absence of typical angiosperm telomeres associated with chromosome ends (Sykorova et al. 2003). With respect to economic value, a number of species are cultivated for their brightly coloured flowers and or fragrance (Beckett 1987) and many represent invasive or potentially invasive species within the tropics, especially on islands in the Pacific Ocean (Mauchamp 1997, Pattison et al. 1998, Space et al. 2003).

The genus *Cestrum* was established by Linnaeus in 1753 (Linnaeus 1753) to accommodate material of two species, *Cestrum nocturnum* L. and *Cestrum diurnum* L. Complete revisions of *Cestrum* have since been undertaken by Dunal (1852) and Francey (1935, 1936). Local treatments exist for the Antilles (Schulz 1909), Guatemala (Gentry and Standley 1974), Nicaragua (D’Arcy 2001), Costa Rica (Standley and Morton 1938), Panama (D’Arcy 1973), Venezuela (Benítez de Rojas and D’Arcy 1998) and Veracruz, Mexico (Nee 1986). As part of this author’s preparation of a revisionary treatment for *Flora Mesoamericana*,eight currently undescribed species were discovered.

The taxonomy of *Cestrum* is complicated by a high proportion of synonyms and unidentified collections. Nee (unpublished) encountered ca. 640 valid names for what he considered to be ca. 150 species. In addition, a relatively high proportion of collections in herbaria remain unidentified to species. Of the 2147 collections examined as part of a taxonomic revision for Flora Mesoamericana, 1003 were identified to species at the time of their accession and 266 were identified subsequent to their incorporation, resulting in 737 (ca. 1/3) collections unidentified to species prior to beginning the Flora Mesoamericana treatment.

## Materials and methods

Two thousand one hundred and forty-seven collections of *Cestrum* from A, BM, F, G, GH, INB, LL, MO, NY, PMA, TEX and US (http://sweetgum.nybg.org/ih/) were examined.Material examined was assigned to ca. 60 morphospecies. Morphospecies were designated on the basis of a visual sort. Morphological characters used for this were the presence of ‘minor’ leaves associated with truncated side branches, leaf shape, texture and size, distribution, inflorescence disposition and size, bract size and shape, calyx and corolla morphology and fruit size, shape and colour. Morphospecies were then examined under a Willd M3C binocular microscope and Planapo lens at X64 to × 400 magnification and a maximum of 117 morphological observations were made for each morphospecies. The morphological characters used included those established by Dunal, Francey and D’Arcy together with axillary bud colour, size and pubescence, position of the petiole, leaf length : width ratio, distribution of the inflorescences on the plant stem, organisation of the flowers in the inflorescence and seed surface morphology. Pubescence was described in terms of hair division, gland association, orientation, shape, size and density. Hair division was classified as simple versus branched and branched hairs were classified as dendritic, stellate etc. Orientation was classified as erect or appressed and shape as straight, curved or crooked. Hair density was somewhat problematic as observations were not taken per square cm but as visual appraisals made using a microscope. Pubescence was arbitrarily classified as dense, sparse or moderate. Modified leaves associated with the inflorescences are described as bracts or bracteoles. Bracts which subtend the pedicel are referred to as bracteoles; those which subtend other parts of the inflorescence are termed bracts.

Following the above detailed examination, the morphospecies were reduced to 47 in number. This reduced number of morphospecies were then associated with validly published names or determined to be undescribed species through a comparison with type material. This resulted in the following eight undescribed species being recognised from the 47 morphospecies.

Conservation Assessments were undertaken using IUCN criteria B, ‘Geographic range,’ in the form of either B1 (extent of occurrence) or B2 (area of occupancy) and A, ‘Population reduction’ (IUCN 2001). Species distributions were plotted using Google Earth and Extent of Occurrence or Area of Occupancy calculated using the grid and ruler tools. In addition, using Google Earth it was possible to assess the nature of the vegetation cover, urbanisation and road proximity at localities, which were used as indicators of plausible future threats. The risks of doing so (Sheppard and Cizek 2009) were considered low with respect to this application of the Google Earth visualisation system.

## Taxonomic treatment

### Cestrum amistadense

A.K.Monro sp. nov.

urn:lsid:ipni.org:names:77116661-1

http://species-id.net/wiki/Cestrum_amistadense

Figs 1 A–D 2 A–C 

#### Diagnosis.

Most similar to *Cestrum langeanum* D’Arcy from which it can be distinguished by the shiny upper leaf surface that is characteristically shrunken around the primary and secondary veins, the raised quaternary and quinternary veins on the upper leaf surface, the glabrous peduncle and the longer inflorescence.

#### Type.

**Panama.** Bocas del Toro: La Amistad Binational Park, La Pata del Cedro camp, ca. 1750 m, 09°04'27"N, 82°44'17W (DMS), 11 Mar 2004, *A. K. Monro & E. Alfaro 4328* (holotype: PMA; isotypes: BM000849623, INB).

#### Description.

Shrubs, 0.4–2.0 m. Leaf–bearing stems drying brown, yellow–brown or olive green, the internodes 25–55 × 1.375–4.0 mm; young stems glabrous. Axillary buds 0.5–1.0 mm, drying dark brown to brown, sparsely pubescent or glabrous, not subtended by a minor leaf. Lamina 90–225 × 26–100 mm, length to width ratio 2.1–3.5, oblong–ovate, ovate, ovate–elliptic, elliptic, or lanceolate, coriaceous, drying yellow–green, brown or dull olive green above, paler below; the upper surface glabrous, primary to quarternary veins, (occasionally primary and secondary only) raised and clearly visible to the naked eye; the lower surface glabrous, primary and secondary, primary to tertiary or primary to quinternary veins raised and clearly visible to the naked eye, secondary veins 6–13 pairs, borne 65–80° to the midrib, irregularly and weakly curved, decurrent, the veinlets visible or not, where visible unbranched or branched; base obtuse to decurrent or asymmetrically acute-cuneate, obtuse, or decurrent; margin entire; apex subcuspidate to cuspidate or acute; petioles 7–25 × 1.25–2.50 mm, drying green, dark brown or yellow–brown, glabrous. Inflorescences 1–3 per herbarium sheet, terminal or subterminal panicles, axillary panicles solitary in each axil, ca. 105 mm long, bearing 7–21 flowers borne in 2–6 clusters, each cluster bearing 3 or 4 flowers; peduncle 25–37 × 1.0–1.5 mm, drying green–brown, yellow–brown or brown, glabrous; bracts 3–100 × 0.75–31.0 mm, leaf–like to bracteole–like; bracteoles 3.0–3.5 mm, linear, glabrous. Flowers pedicellate, the pedicels 0.50–0.75 mm; calyx 3.25–4.50 × 2.0–2.25 mm, the outer surface glabrous, the lobes 5, 0.50–0.675 mm, erect; corolla pale purple to lilac, 26–34 mm long, the tube 26–28 mm long, 3.0–3.5 mm in diameter at the mouth, ca. 1.25 mm in diameter at the base, glabrous, the lobes 5, 6.5–8.0 mm long; stamens 5, the filaments 23.5–26.0 mm long, equal, adnate for 19.0–20.5 mm of their length, glabrous, a lobe–like appendage present at insertion point, the anthers ca. 1 × 0.675–0.750 mm; style 24.5–26.0 mm, the stigma 0.375–0.50 × 0.75–1.25 mm. Infructescences 25-80 mm long, bearing 5–12 fruit; fruiting calyx 3.5–4.0 × 7–8 mm; fruit not seen.

**Figure 1.**
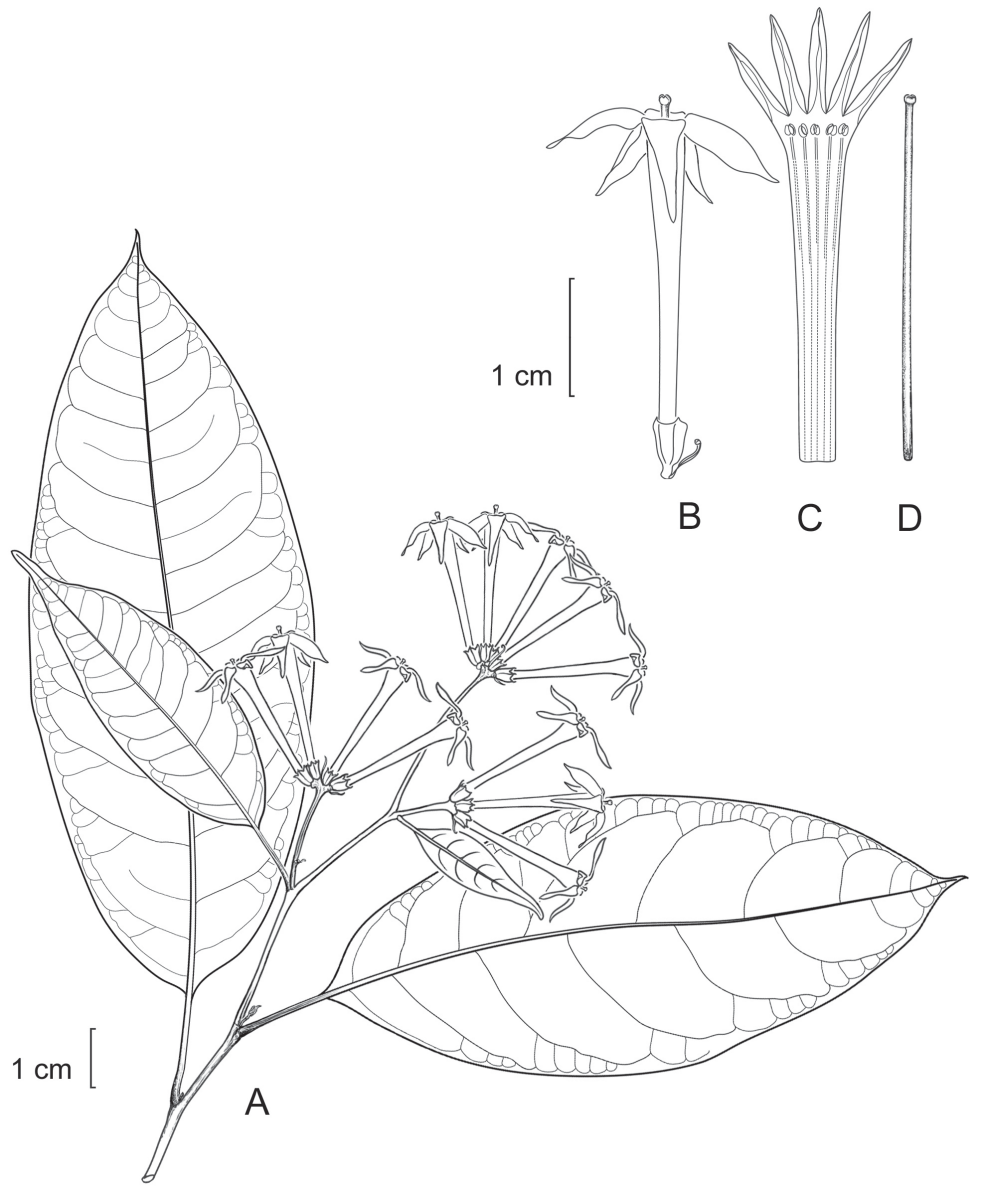
*Cestrum amistadense*A.K.Monro **A** Flowering stem **B** Flower **C** Longitudinal section through the corolla showing stamen arrangement **D** Style. Drawn from *A.K. Monro & E. Alfaro 4328*(BM).

**Figure 2.**
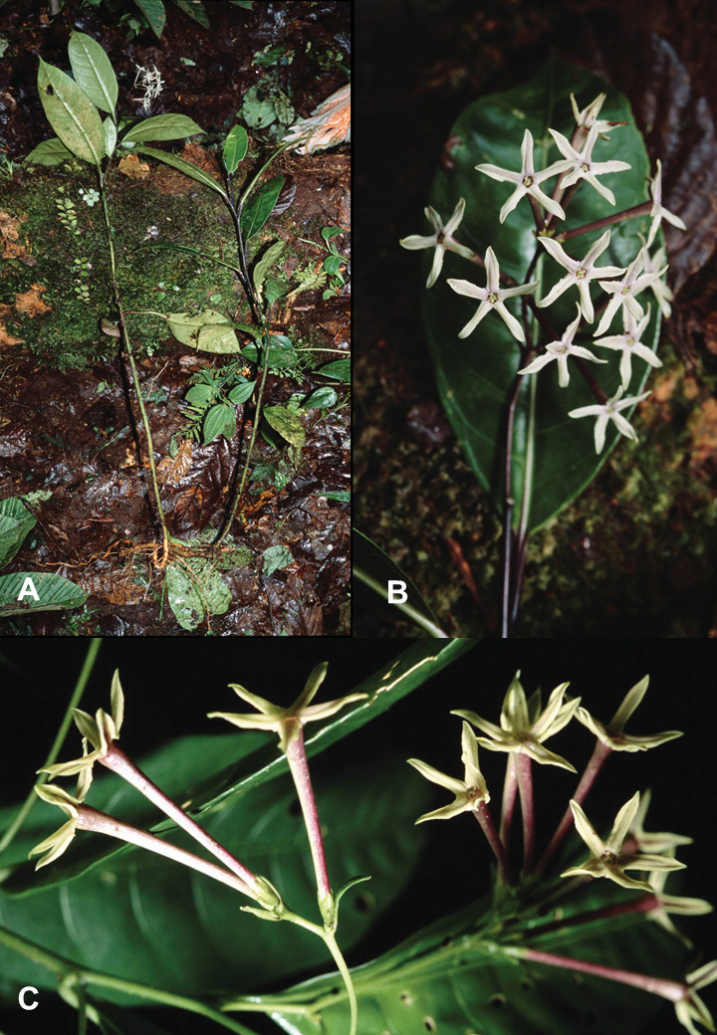
*Cestrum amistadense*A.K.Monro **A** Habit. **B**. Inflorescence **C** Flowers. *A.K. Monro & E. Alfaro 4415* (Photo A.K.Monro 2004).

#### Etymology.

From the locality of the holotype, La Amistad Binational Park in Costa Rica and Panama.

#### Distribution.

Premontane, montane, cloud and oak forest from 900 m to 2100 m. Collection notes indicate that this species is restricted to undisturbed or ‘high’ forest. Existing collection localities suggest that *Cestrum amistadense* is distributed over an area of ca. 7,360 km^2^ of the Fila Costeña and the Talamanca Mountains in eastern Costa Rica and western Panama (Google Earth, accesssed April 21 2011, images from 2011).

#### Discussion.

Of the six known collections of *Cestrum amistadense*, none had been previously determined to species. Comparison of the holotype and paratype material with type specimens from the herbaria listed in the Materials and methods section recovered *Cestrum amistadense* as most similar to *Cestrum langeanum* D’Arcy and *Cestrum longiflorum* Ruiz & Pav. It can be distinguished from those speciesby the presence and distribution of pubescence, leaf surface, venation and inflorescence morphology as summarised in Tables 1 and 2.

**Table 1.** 

Characters	*Cestrum amistadense*	*Cestrum langeanum*
Upper leaf surface morphology	shiny and shrunken around primary and secondary veins	matte, not appearing shrunken around primary and secondary veins
Upper leaf surface quarternary and quinternary veins raised	yes	no
Peduncle pubescence	glabrous	pubescent
Inflorescence length in flower	ca. 105 mm	21–55 mm

**Table 2.** 

Characters	*Cestrum amistadense*	*Cestrum longiflorum*
Lower leaf surface pubescence	glabrous	pubescent
Upper leaf surface quarternary and quinternary veins raised	yes	no
Peduncle pubescence	glabrous	pubescent
Bracteole length	3.0–3.5 mm	2.5 mm

#### Conservation status.

Using IUCN criteria (IUCN 2001) *Cestrum amistadense* is considered Vulnerable based on subcriteria B: Extent of Occurrence <20,000 km^2^ (B1), a severely fragmented range and small number of collection localities (B1a) and continuing decline in the area of habitat due to the conversion of forest to agricultural land (B1b).

#### Paratypes.

COSTA RICA. Limón: Cantón de Talamanca, Bratsi, Amubri, Alto Lari, Kivut, siguiendo la fila divisoria entre Ríos Lari y Dapari, cuenca superior de ambos, 09°22'45"N, 83°06'15"W (DMS), 1900 m, 24 Mar 1992, *B. Hammel 5474* (INB, MO); Puntarenas: Cantón de Osa, Río Piedras Blancas, junto a la casa, faldas Cerro Anguciana, Fila Cruces, 900 m, 08°49'02"N, 83°11'23"W (DMS), 9 Dec 1993, *R. Aguilar, D. Gómez, M. Grayum & B. Hammel 2732* (BM, INB). PANAMA. Bocas del Toro-Chiriquí border: end of Río Palo Alto road to Chiriquí border with Bocas del Toro, near peak of Cerro Macho, 08°49'N, 082°23'W (DMS), 2200 m, 20 Nov 1978, *B. Hammel 5799* (MO); La Amistad Binational Park, Quebrada by Campamiento Lucio, 09°05'03"N, 082°45'44"W (DMS), 1850 m, Mar 16 2004, *A. K. Monro & E. Alfaro 4415* (INB, MO, PMA); Chiriquí: end of road past Palo Alto northeast of Boquete 2100 m, 8 Feb 1979, *B. Hammel 6062* (MO); trail to Cerro Pate Macho, headwaters of the Río Palo Alto, above Palo Alto, 1700–2100 m, 08°47'N, 82°22'W (DMS), 24 Apr 1982, *S. Knapp & R. Schmalzel 4766* (MO).

### Cestrum contrerasianum

A.K.Monro sp. nov.

urn:lsid:ipni.org:names:77116662-1

http://species-id.net/wiki/Cestrum_contrerasianum

Fig. 3 A–C 

#### Diagnosis.

Most similar to *Cestrum formosum* C.V. Morton from which it can be distinguished by its more compact inflorescences, bearing flowers on shorter pedicels with pubescent calyces and longer corolla tubes.

#### Type.

**Guatemala.** Baja Verapaz: Union Barrios, 15°11'01"N, 090°12'16"W (DMS), 1630 m,11 Mar 1972, *E. Contreras 11234* (holotype: F-1942896; isotype: MO).

#### Description.

Shrubs, 1–7 m. Leaf–bearing stems drying pale red–brown or tan, the internodes 2–34 × 1.5–3.5 mm; young stems glabrous, occasionally sparsely pubescent, where pubescent the hairs 0.375 mm, simple, appressed, crooked and frequently glandular. Axillary buds 1.25–2.0 mm, frequently absent, drying dark brown, densely pubescent and glandular, not subtended by a minor leaf. Lamina 13–135 × 6–42 mm, length to width ratio 1.5–3.6, obovate, elliptic to ovate, coriaceous, drying olive green, brown or yellow–green above; the upper surface glabrous or very sparsely pubescent, the hairs 0.125 mm, simple, weakly appressed, crooked, occasionally sparsely glandular, primary only or primary and secondary veins clearly visible to the naked eye, only the primary veins raised; the lower surface glabrous, sparsely punctate glandular, primary only, primary and secondary or primary to tertiary veins clearly visible to the naked eye, primary only or primary and secondary veins raised, secondary veins 3–6 pairs, borne 45–75° to the midrib, weakly curved, the veinlets not visible; base decurrent, cuneate, or acute, occasionally asymmetrically so; margin entire; apex acute or subcuspidate; petioles borne on a woody or crescent-shaped spur or regularly from the stem, 5–66 × 0.75–1.25 mm, drying dark purple, dark brown, yellow–green or green, glabrous, occasionally sparsely punctate glandular. Inflorescences 6–13 per herbarium sheet, terminal, subterminal or borne along the full length of the leaf–bearing portion of the stem, axillary panicles solitary in each axil, 25–60 mm long, bearing 1–16 flowers in 1–3 fascicle–like clusters, each cluster bearing 1–5 flowers; peduncle 1.5–20.0 × 0.75–1.75 mm, yellow–brown through orange–brown to dark brown, glabrous tosparsely or very sparsely pubescent, the hairs 0.25 mm, simple, curved or straight, occasionally glandular; bracts 18–43 × 9–13 mm, leaf–like; bracteoles 4–22 mm, ovate, linear, glabrous or sparsely pubescent. Flowers pedicellate, the pedicels 0.5–1.5 mm; calyx 5–6.5 × 2.0–2.5 mm, the outer surface glabrous, the lobes 4–6, 1.25–2.5 mm long, erect; corolla yellow–green or pale yellow to yellow, 4–26 mm long, the tube 9.0–21.5 mm long, 2.5–4.25 mm in diameter at the mouth, 0.75–1.50 mm in diameter at the base, glabrous, the lobes 4–6, 3.5–5.5 mm long; stamens 5, the filaments 16–19 mm, equal, adnate for 6–9 mm, pubescent from insertion point to the base, with a 1–lobed keel–shaped or reduced knee–like appendage present at insertion point, the anthers 1.0–1.5 × 0.675–1.0 mm; style 11–18 mm, the stigma 0.5–1.0 × 1–2 mm. Infructescences 10–65 mm long, bearing 2–6 fruit; fruiting calyx 3.5–7.5 × 6–9 mm; fruit 7–10 × 4–7 mm, subglobose to oblongoid, white when ripe. Seeds 1–10, 3–6 × 1.5–3.5 × 2–3 mm.

**Figure 3.**
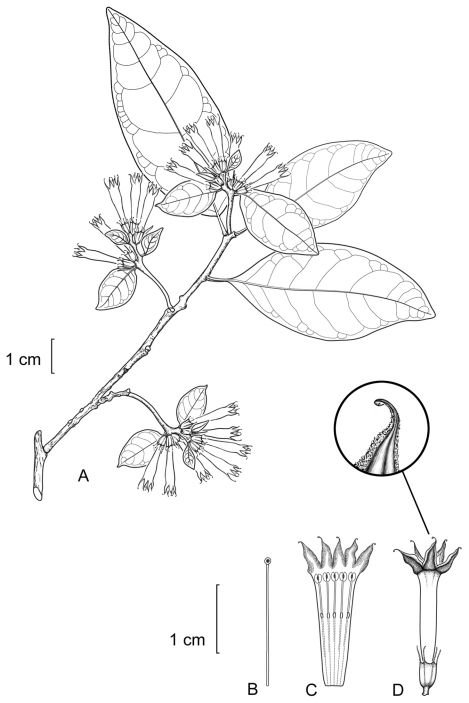
*Cestrum contrerasianum*A.K.Monro **A** Flowering stem **B** Style **C** Longitudinal section through the corolla showing stamen arrangement **D** Flower with apex of corolla lobe highlighted. Drawn from *E. Contreras 11234* (F).

#### Etymology.

After Elias Contreras, Guatemalan plant collector and botanist who collected the type and two of the paratype collections of this species.

#### Distribution.

Montane or cloud forest, often undisturbed forest. Existing collection localities suggest that *Cestrum contrerasianum* is distributed along the Pacific drainage of central Guatemala (Baja Verapaz, El Quiche, Huehuetenango) and southeastern Mexico (Chiapas) in an area encompassing ca. 13,400 km^2^ (Google Earth, accessed 10 Dec 2010).

#### Discussion.

Of the sixteen known collections of *Cestrum contrerasianum*,ten were previously determined as *Cestrum aurantiacum* Lindl. Comparison of the holotype and paratype material with type specimens from the herbaria listed in the Materials and methods section recovered *Cestrum contrerasianum* as most similar to *Cestrum formosum* C.V. Morton. It can be distinguished from those species on the basis of inflorescence number and morphology and flower and fruit morphology as summarised in Tables 3 & 4.

**Table 3.** 

Characters	*Cestrum contrerasianum*	*Cestrum aurantiacum*
Inflorescence no. per herbarium sheet	6–13	1–4
Inflorescence length	25–60 mm	58–192 mm
Inflorescence panicle type	compact	not compact
Pedicel length	0.5–1.5 mm	0.5–2.5 mm
Calyx pubescence	sparsely pubescent	glabrous
Fruit size	7–10 × 4–7 mm	ca. 12 × 9.5–10.0 mm

**Table 4.** 

Characters	*Cestrum contrerasianum*	*Cestrum formosum*
Inflorescence panicle type	compact	not compact
Pedicel length	0.5–1.5 mm	2–5 mm
Calyx pubescence	sparsely pubescent	glabrous
Calyx lobe length	1.25–2.5 mm	0.50–0.675
Corolla length	14–26 mm	10–14 mm

#### Conservation status.

Using IUCN criteria (IUCN 2001) *Cestrum contrerasianum* is considered Vulnerable (VU B1ab(iii)) based on subcriteria B1 based on an Extent of occurrence <20,000 km^2^, a severely fragmented range and continuing decline in the area of habitat.

#### Paratypes.

GUATEMALA. Baja Verapaz: Union Barrios, W of km 154, 15°11'01"N, 090°12'16"W (DMS), 1600 m, Aug 17 1975, *C. L. Lundell & E. Contreras 19671* (F); Niño Perdido, La Cumbre de San José Espinero, 15°07'54"N, 090°09'46"W (DMS), 18 May 1977, *C. L. Lundell & E. Contreras 20913* (CAS, F). Huehuetenango: Cerro Huitz, between Mimanhuitz and Yulhuitz, Sierra de los Cuchumatanes, 15°51'18"N, 091°19'28"W (DMS), 1500–2600 m, 14 Jul 1942, *J. A. Steyermark 48657* (F); vicinity of Nucapuxlac, 2500 m, 17 Jul 1942, *J. A. Steyermark 48943* (AA, F). El Quiché: about 7 km SWW of Nebaj, 2200 m, Jun 22 1964, *E. Contreras 5088* (LL). MEXICO. Chiapas: Municipio of San Cristóbal las Casas, 2 miles northeast of Highway 190 along the old road to Huistán, 2300 m, 22 Jan 1965, *D. E. Breedlove & P. Raven 8294* (US); Municipio of Tenejapa, along the river of Chik Ha', barrio of Yashanal, paraje of Matsab, 1700 m, 17 Jul 1965, *D. E. Breedlove 11109* (F); Municipio La Trinitaria, Lagos de Montebello, 42 km northeast of La Trinitaria, 16°07'05"N, 091°42'27"W (DMS), 1300 m, 23 Oct 1971, *D. E. Breedlove & R. F. Thorne 21152* (DS); Municipio of Comitán de Dominguez, 5 km north of Highway 190 on a logging road from Laguna Chamula microwave station, 2400 m, 15 Oct 1976, *D. E. Breedlove & B. Bartholomew 40775* (DS); Municipio of La Independencia, third ridge along logging road from Las Margaritas to Campo Alegre, 2300 m, 24 Oct 1976, *D.E. Breedlove 41079* (DS); Municipio of Comitán de Dominguez, Laguna Chamula microwave station, 4 km southwest of Highway 190 between Comitán and Amatenango del Valle, 2500 m, 6 Nov 1981, *D. E. Breedlove & G. Davidse 54848* (CAS); San Felipe village, near Ciudad San Cristobal las Casas, Mount Ecatepec, 3 miles W of village, 2400 m, Mar 31 1949, *M. C. Carlson 1614* (F); Municipio of San Cristóbal las Casas, a 200 m del Paraje K'ostic, 16°43'20"N, 092°31'29"W (DMS), 2300 m, 5 Sep 1994, *A. Chamé & A. Luna 352* (CAS); temperate land, 1864–70, *A. B. Ghiesbreght 837* (GH); Municipio of San Cristóbal las Casas, Cerro El Extranjero, 16°39'39"N, 092°34'26"W (DMS), 2350 m, 26 Mar 1992. *M. González E., P. F. Quintana A., M. Martínez I. & N. Ramírez M. 1769* (CAS).

### Cestrum darienense

A.K.Monro sp. nov.

urn:lsid:ipni.org:names:77116663-1

http://species-id.net/wiki/Cestrum_darienense

Fig. 4 A–D 

#### Diagnosis.

Most similar to *Cestrum morae* Hunz. from which it can be distinguished by its thinner, more delicate stems, the lower number of secondary veins in the lamina, the ascendant and shorter inflorescences bearing flowers with shorter corolla tubes and longer corolla lobes.

#### Type.

**Panama.** Darién: Cerro Pirre, valley between between Pirre and next most southerly peak, sloping hillside, 07°40'N, 077°42'W (DMS), 1250–1300 m, *J.P. Folsom, R. L. Hartman & R. L. Dressler 4415* (holotype: MO-2620728).

#### Description.

Shrub, occasionally lax, 1.0–2.5 m. Leaf–bearing stems grey–brown, the internodes 16–95 × 1.25–2.75 mm; young stems moderatley pubescent, the hairs 0.125–<0.25 mm, simple, weakly appressed, curved, glandular. Axillary buds 0.675–1.0 mm, red–brown, densely pubescent, not subtended by a minor leaf. Lamina 55–160 × 22–83 mm, length width ratio 1.86–3.0, broad rhombic–elliptic, rhombic–obovate, ovate–rhombic, obovate, rhombic, chartaceous, brown–green to brown; the upper surface glabrous, primary and secondary, primary to tertiary veins clearly visible to the naked eye, primary, primary and secondary raised; the lower surface sparsely pubescent, the hairs ca. 0.125 mm, simple, sessile to subsessile, glandular, weakly appressed, straight, orange–brown, primary to quarternary, primary to tertiary veins clearly visible to the naked eye, primary and secondary, primary to tertiary veins raised, the veinlets visible, unbranched, secondary veins 3–7 pairs, 30–45° to the midrib, straight, weakly curved; base subcordate, asymmetrical, obtuse, cordate-cuneate, cuneate; margin entire to irregularly entire; apex cuspidate, acuminate, subacuminate; petioles bottle-shaped, 3.5–8.0 × 1.375–2.0 mm, dark brown, moderatley pubescent, sparsely pubescent or glabrous, the hairs 0.125–0.25 mm. Inflorescences 2–13 per herbarium sheet, axillary, solitary in each axil, borne along the full length of leaf–bearing stem, 35–48 mm long, bearing 4–6 flowers in a panicle, the bracts forming a loose involucre around 1 or 2 clusters of flowers, each cluster bearing 3–5 flowers; peduncle 3.5–13.0 × 0.5–1.0 mm, dark brown to brown, moderatley pubescent or sparsely pubescent, the hairs 0.125–0.250 mm, glandular; bracts 3.5–10.0 × 2.0–5.5 mm, leaf–like; bracteoles 6–10 mm, ovate, obovate, rhombic, moderatley pubescent. Flowers subsessile, the pedicels < 0.25 mm; calyx 2.75–3.25 × 1.25–1.75 mm, the outer surface glabrous, the lobes 5, 0.50–0.675 mm long, erect; corolla white, 22–30 mm long, the tube 18.5–24.0 mm long, 1.5–2.0 mm in diameter at the mouth, 0.675–1.0 mm at the base, glabrous, the lobes 5, 7–9 mm long; stamens 5, the filaments 15–22 mm, equal, adnate for 13.5–19.5 mm, glabrous, with a lobe–like appendage present at insertion point, the appendage pubescent, the anthers 0.75 × 0.675 mm; style 17.0–19.5 mm, the stigma 0.675 × 0.75 mm. Infructescences and fruit not seen.

**Figure 4.**
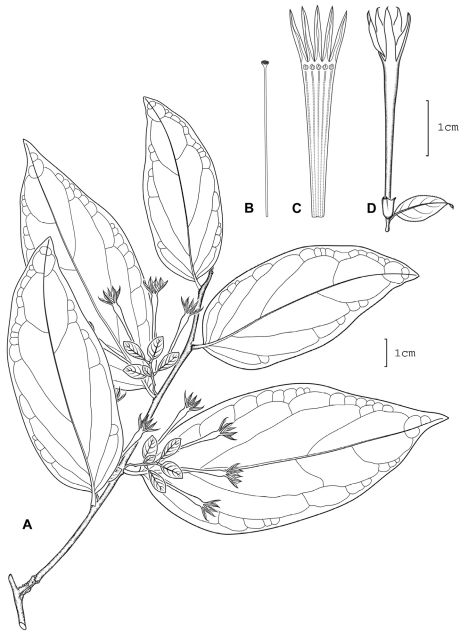
*Cestrum darienense*A.K.Monro **A** Flowering stem **B** Style **C** Longitudinal section through the corolla showing stamen arrangement **D** Flower. Drawn from *J.P. Folsom, R.L. Hartman & R.L. Dressler 4415* (MO).

#### Etymology.

This species is named after the Darien province, locality of the type and paratype collections.

#### Distribution.

*Cestrum darienense* is known from two localities in Cerro Pirre and whilst none of the collection labels indicate a forest type, altitude information would suggest that this would be cloud forest. Cerro Pirre covers an area of ca. 50 × 25 km. Using collection label data and Google Earth (accesssed June 7, 2011; images from 2003) the Extent of Occurrence for this species is calculated to be ca. 280 km^2^.

#### Discussion.

Of the three collections of *Cestrum darienense* seen, none had been previously determined to species prior to this study. A comparison of the holotype and paratype material with type specimens from the herbaria listed in the Materials and methods section recovered *Cestrum darienense* as most similar to *Cestrum gilliae* A.K. Monro and *Cestrum morae* Hunz. Together with *Cestrum langeanum* D’Arcy, *Cestrum darienense*, *Cestrum gilliae* and *Cestrum morae* form a coherent morphological and geographical grouping within the genus of species from western Panama and the Chocó in Colombia and Ecuador characterised by broad, nearly three-veined leaves and determinate inflorescences. *Cestrum darienense* can be distinguished from *Cestrum morae* and *Cestrum gilliae* on the basis of stem, leaf, inflorescence and flower morphology as summarised in Tables 5 & 6. The Cerro Pirre mountain range has been noted as a locality for many endemic plant and animal species; this has been attributed to it being a moist forest refugium during dry periods of the Pleistocene (Haffer 1967).

**Table 5.** 

Characters	*Cestrum darienense*	*Cestrum morae*
Stem diameter at position of the inflorescences	1.0–2.5 mm	4–6 mm
Leaf lamina width	22–83 mm	105–198 mm
Secondary vein number	3–7 pairs	14–20 pairs
Inflorescence length	35–48 mm	220–320 mm
Inflorescence disposition	spreading to 45°	lax, pendent
Corolla length	22–30 mm	37–39 mm
Corolla lobes	7–9 mm	6–7 mm
Stamen length	15–22 mm	ca. 30 mm
Filament appendage	present, lobe–like	absent

**Table 6.** 

Characters	*Cestrum darienense*	*Cestrum gilliae*
Leaf lamina length	55–160 mm	135–285 mm
Secondary vein no.	3–7	9–15
Inflorescence length	35–48 mm	150–210 mm
Peduncle diameter (basal 1/3)	0.5–1.0 mm	2–3 mm
Calyx length	2.75–3.75 mm	4–6 mm
Corolla lobe length	7–9 mm	4.0–4.5 mm

#### Conservation status.

Using IUCN criteria (IUCN 2001), *Cestrum darienense* is considered to be Near Threatened. The Extent of Occurrence is calculated to be ca. 280 km^2^ (Criteria B1 <5,000km^2^) and there are only two known localities (Criteria B1a ≤5, Endangered). No decline in geographic range or fragmentation of the habitat has been observed, however, and Cerro Pirre is located within the Darién Biosphere Reserve, a UNESCO World Heritage Property. The Darién is vulnerable to factors which may result in future deforestation, e.g. economic development associated with the resolution of the armed conflict in neighbouring Colombia and or an end to narco-trafficking. Any associated decline in geographic range or fragmentation would result in this species being assessed as Endangered.

#### Paratypes.

PANAMA. **Darién:** Serranía de Pirre, trail ca. 1 mi SSW of Cerro Pirre summit, 07°56'N, 077°42'W (DMS), 1200 m, 15 Jul 1977, *R. L. Hartman, J. P. Folsom & R. L. Dressler* 4655 (MO); Cerro Pirre, along stream between Pirre and next southern peak, 07°40'N, 077°42'W (DMS), 10–20 Jul 1977, *J. P. Folsom* 4383 (MO).

### Cestrum gilliae

A.K.Monro sp. nov.

urn:lsid:ipni.org:names:77116664-1

http://species-id.net/wiki/Cestrum_gilliae

Fig. 5 A–D 

#### Diagnosis.

Most similar to *Cestrum morae* Hunz. from which it can be distinguished by the fewer secondary nerves, the ascendant inflorescences bearing flowers along a greater proportion of its length, shorter bracteoles and flowers and filaments with a 2-lobed appendage towards its base.

#### Type.

**Panama.** Darien: Rancho Frío to summit of Cerro Pirre, 07°51'N, 077°42'W – 07°58'N, 077°43'W (DMS), 500–1140 m, 30 Mar 1985, *W. G. D'Arcy & G. McPherson 16210* (holotype: MO-4407602).

#### Description.

Robust herb to shrub 1.5–2.0 m. Leaf–bearing stems dark grey–brown, the internodes 15–43 × 4–6 mm; young stems regularly or sparsely pubescent, the hairs 0.25–0.75 mm, branched, dendritic, erect, crooked, not glandular. Axillary buds 1.5–2.5 mm, brown, densely pubescent, not subtended by a minor leaf. Lamina 135–285 × 32–124 mm, length width ratio 2.0–4.2, oblong–elliptic, obovate, elliptic, or oblong–obovate, chartaceous to subcoriaceous, brown or dull green, the upper surface glabrous, sparsely pubescent at the base, the hairs 0.375 mm, branched, erect, crooked dendritic, primary and secondary veins clearly visible to the naked eye, primary or primary and secondary raised; lower surface sparsely pubescent on nerves, the hairs 0.125–0.50 mm, branched, glandular, erect, crooked–dendritic, orange–brown; primary and secondary or primary to tertiary veins clearly visible to the naked eye, primary and secondary veins raised, the veinlets not visible, secondary veins 9–15 pairs, 30–45° to the midrib, straight, apically curved; base obtuse, cuneate, asymmetrical, obtuse-cuneate; margin entire; apex subcuspidate to cuspidate; petioles 12–23 × 1.75–3.0 mm, brown to very dark brown, moderatley pubescent when young, becoming glabrous with age or always glabrous, the hairs 0.25–0.75 mm. Inflorescences 1–3 per herbarium sheet, terminal, indeterminate, solitary, clustered towards the branch tips, 150–210 mm long, bearing 27–36 flowers borne in panicles of 10–16 clusters of flowers, each cluster bearing 1–3 flowers; peduncle ca. 30 × 2–3 mm, brown to very dark brown, sparsely pubescent, the hairs 0.375–0.675 mm, branched, dendritic, eglandular; bracts 7–41 × 1.5–12 mm, leaf–like; bracteoles 6–7 mm, narrow oblong to linear, sparsely pubescent; flowers subsessile or pedicellate, where pedicellate the pedicels 0.25–0.50 mm; flowers yellow–green or white, becoming purple blue; calyx 4–6 × 2.25–3.0 mm, the outer surface glabrous, the lobes 3 or 5, 0.75–1.75 mm, erect or spreading; corolla 26–30 mm long, the tube 23–27 mm long, 2–3 mm in diameter at the mouth, 1–2 mm at the base, glabrous, the lobes 5, 4.0–4.5 mm long; stamens 5, the filaments 22–23 mm long, equal, adnate for 16.5–17.5 mm, pubescent, with a bilobed appendage present at insertion point, the anthers 1.25 × 1.0 mm; style 22–25 mm, the stigma 0.50–0.75 × 0.675–1.0 mm. Infructescences ca. 125 mm long, bearing ca. 14 fruit; calyx 6 × 7.0–7.5 mm; fruit immature, 5–6 × 5 mm, purple–black when ripe. Seeds not seen.

**Figure 5.**
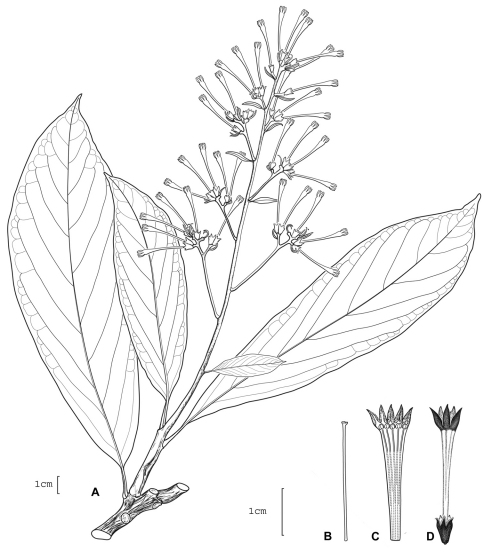
*Cestrum gilliae*A.K.Monro **A** Flowering stem **B** Style **C** Longitudinal section through the corolla showing stamen arrangement **D** Flower. Drawn from *W.G. D’Arcy & G. McPherson 16210* (MO).

#### Etymology.

This species is named in honour of Gill Stevens (née Douglas, born 1965), botanist, colleague and close friend who died during the preparation of this manuscript. Gill, an algologist by training, helped pioneer the use of amateur groups in the collection of biodiversity data in the UK.

#### Distribution.

*Cestrum gilliae* is known from three cloud forest localities within the Cerro Pirre mountain range. Cerro Pirre covers an area of ca. 50 × 25 km. Using collection label data and Google Earth (accesssed June 6, 2011; images from 2003) the Extent of Occurrence is calculated to be ca. 260 km^2^.

#### Discussion.

Of the three collections of *Cestrum gilliae* seen, only one had been previously determined to species, as *Cestrum langeanum* D’Arcy. A comparison of the holotype and paratype material with type specimens from the herbaria listed in the Materials and methods section recovered *Cestrum gilliae* most similar to *Cestrum morae* Hunz. and to a lesser extent *Cestrum langeanum* D’Arcy (see Discussion for *Cestrum darienense* above). For this reason *Cestrum gilliae* is contrasted to both *Cestrum morae* and *Cestrum langeanum* on the basis of habit and inflorescence and flower morphology as summarised in Tables 7 & 8 respectively.

**Table 7.** 

Characters	*Cestrum gilliae*	*Cestrum morae*
Habit	robust herb or shrub	shrub, tree or vine
Secondary vein number	9–15 pairs	14–20 pairs
Inflorescence disposition	spreading to 45°	lax, pendent
Flower disposition	flowers borne along the apical 2/3 of the inflorescence length	flowers borne along the apical 1/3 to 1/8 of the inflorescence length
Bracteole length	6–7 mm	11–15 mm
Corolla length	26–30 mm	37–39 mm
Corolla lobe length	4.0–4.5 mm	6–7 mm
Stamen length	22–23 mm	ca. 30 mm
Filament adnation	16.5–17.5 mm of its length	24–25 mm of its length
Filament appendage	bilobed	absent

**Table 8.** 

Characters	*Cestrum gilliae*	*Cestrum langeanum*
leaf shape	oblong–elliptic, elliptic, obovate, oblong–obovate	narrowly ovate, narrowly elliptic, elliptic
Leaf texture	chartaceous to subcoriaceous	coriaceous
Secondary vein angle of insertion	30–45°	60–85°
Leaf apex	subcuspidate to cuspidate	acute to attenuate
Inflorescence length	150–210 mm	21–70 mm
Calyx dimensions	4–6 × 2.25–3.0 mm	3.25–4.5 × 1.75–2.25 mm
Corolla lobe length	4.0–4.5 mm	5.5–7.0 mm
Filament appendage	bilobed	absent

#### Conservation status.

Using IUCN criteria (IUCN 2001) *Cestrum gilliae* is considered to be Near Threatened under IUCN (2001) criteria. The Extent of Occurrence is calculated to be ca. 260 km^2^ (Criteria B1, <5,000km^2^) and there are only three known localities (Criteria B1a, ≤5). No decline in geographic range or fragmentation of the habitat has been observed and Cerro Pirre is located within the Darién Biosphere Reserve, a UNESCO World Heritage Property. The Darién is, however, vulnerable to factors (see Conservation Status above for *Cestrum darienense*) and any decline in geographic range or fragmentation would result in this species being assessed as Endangered. For this reason *Cestrum gilliae* is considered Near Threatened.

#### Paratypes.

PANAMA. Darién: Cerro Pirre ridge top near Rancho Plastico, 07°57'N, 077°42'W (DMS), 1200 m, 10–20 Jul 1977, *J. P. Folsom* 4253 (MO); Cuasí–Cana Trail between Cerro Campamiento and La Escalera to ‘Paramo' east of Tres Bocas, 07°44'N, 077°44'W – 07°46'N, 077°47'W (DMS), 30 Apr 1968, *J. H. Kirkbride & J. A. Duke* 1277 (MO).

### Cestrum haberii

A.K.Monro sp. nov.

urn:lsid:ipni.org:names:77116665-1

http://species-id.net/wiki/Cestrum_haberii

Fig. 6 A–D 

#### Diagnosis.

Most similar to *Cestrum poasanum* Donn.Sm. from which it can be distinguished by the broader leaves and the shorter pedunculate or sessile inflorescences bearing flowers with usually shorter pedicels.

#### Type.

**Costa Rica.** Cantón de Puntarenas, Monteverde, Pacific slope wet forest, 10°18'N, 84°48'W (DMS), 1400–1500 m, 12 Mar 1992, *W. A. Haber 11049* (holotype: MO-3930789).

#### Description.

Trees or shrubs, where shrubs occasionally lax, 1–5 m. Leaf–bearing stems drying pale brown to dark brown, grey–brown, green–grey or grey, the internodes 7–60 × 1.5–8.0 mm; young stems glabrous, sparsely pubescent or moderatley pubescent, where pubescent the hairs 0.25–0.50 mm, branched, erect, dendritic, eglandular. Axillary buds 0.75–2.50 mm, drying pale brown to dark brown or red–brown or dark grey–brown, densely pubescent to regularly pubescent, not subtended by a minor leaf. Lamina 55–250 × 19–110 mm, length width ratio 1.8–3.3(4.1), ovate–oblong, oblong–obovate, ovate, obovate, or oblong, chartaceous to subcoriaceous, drying olive green, brown or yellow–brown, upper surface glabrous or sparsely pubescent, minutely pusticulate, where pubescent the hairs 0.250–0.375 mm, branched or simple, appressed, dendritic where branched, straight where simple; primary and secondary veins clearly visible to the naked eye, the primary and secondary raised; lower surface sparsely to very sparsely pubescent, the hairs 0.25–0.50 mm, branched, erect, weakly appressed, dendritic where branched, glandular where simple, dark walled, orange–brown to brown in colour; primary to quarternary veins clearly visible to the naked eye, primary and secondary only or primary to tertiary veins raised, veinlets not visible, secondary veins 6–13 pairs, borne 45–75° to the midrib, curved to weakly curved, strongly ascending; base decurrent or cuneate or asymmetrically obtuse / decurrent or cuneate / decurrent; margin entire, very irregularly and weakly crenate; apex subcuspidate, cuspidate or acute; petioles frequently borne on a crescent shaped short woody spur, 11–43 × 0.75–2.25 mm, brown to very dark brown, sparsely pubescent or glabrous, where pubescent the hairs 0.125–0.50 mm. Inflorescences 3–9 per herbarium sheet, axillary and terminal, solitary, borne along the full length of the leaf–bearing stem, 25–300 mm, panicle occasionally branched to its base, bearing 7–120 flowers in 2–25 clusters, each cluster bearing 1–8 flowers; sessile or peduculate, where pedunculate the peduncle 2–25 × 0.675–1.25 mm, brown to dark brown, densely pubescent or moderatley pubescent, the hairs 0.250–0.675 mm, branched, dendritic, eglandular; bracts (1.5)10–46 × 1.25–14.0 mm, leaf–like; bracteoles 0.5–5.0 mm, frequently caducous, linear, moderatley pubescent or densely pubescent. Flowers pedicellate or subsessile, where pedicellate the pedicels 0.375–0.750 mm; flowers yellow–green, pale yellow, cream, white or dull pink, the lobes occasionally lilac coloured, nocturnally fragrant; calyx 2.75–6.0 × 1.675–2.0 mm long, the tube 11–21 mm long, the outer surface glabrous, the lobes 3–5(6–7), 0.50–2.0 mm, erect; corolla 15–24 mm, 2.0–3.75 mm in diameter at the mouth, 0.5–1.5 mm at the base, glabrous, the lobes 5, 2.5–4.5 mm; stamens 5, the filaments 13.5–19.0 mm long, subequal, adnate for 12.0–16.5 mm, with a lobe–like or bilobed appendage present at insertion point, pubescent from appendage to base, anthers 0.675–1.250 × 0.675–1.0 mm; style 14–20 mm, the stigma 0.675–0.750 × 0.675–1.0 mm. Infructescences 32–100 mm long, bearing 3–15 fruit; fruiting calyx 3.0–4.5 × 4–6 mm; fruit 7.5–10.0 × 5–8 mm, white or cream when ripe. Seeds 7–10, 2.5–4.5 × 1.5–2.0 × 1.5–2.0 mm, the surface minutely verrucate.

**Figure 6.**
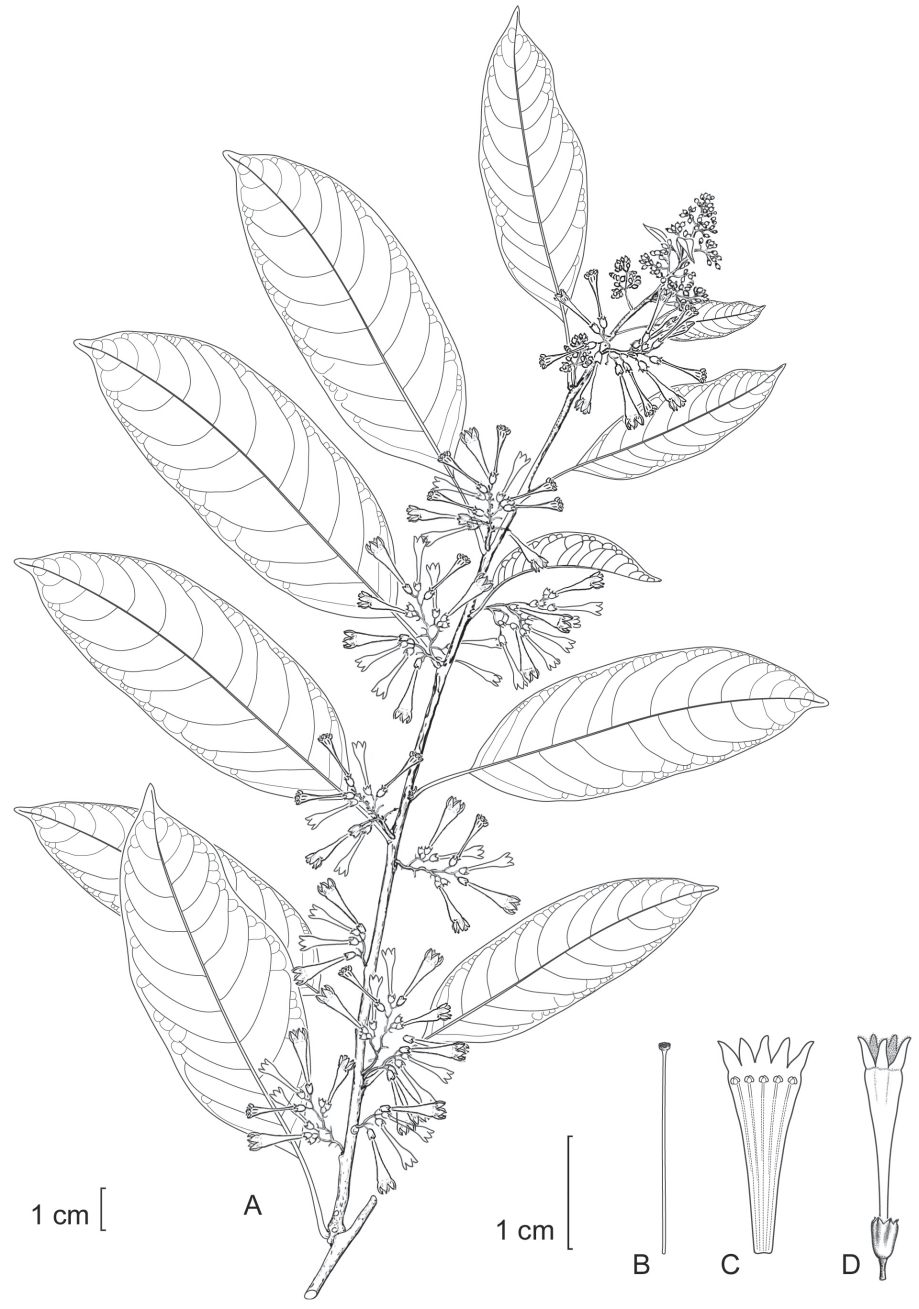
*Cestrum haberii*A.K.Monro **A** Flowering stem **B** Style **C**. Longitudinal section through the corolla showing stamen arrangement **D** Flower. Drawn from *W.A. Haber 11049* (MO).

#### Etymology.

This species is named after William Haber, US botanist (1946–), who collected the holotype and six of the paratype collections.

#### Distribution.

Wet, cloud and montane forest from (100) 900 to 2200 m. Collection notes indicate that this species is known from undisturbed and disturbed forest. Existing collection localities suggest that the species’ Extent of Occurrence is 30,950 km^2^ running along the Pacific coast of Costa Rica (Guanacaste, Alajuela, Heredia, Cartago, San José, Puntarenas) and Panama (Chiriquí) (Google Earth, accessed Dec 16 2010, images 2001, 2006).

#### Discussion.

Of the 29 known collections of *Cestrum haberii* most had previously been determined as *Cestrum poasanum* Donn. Sm. A comparison of the holotype and paratype material with type specimens from the herbaria listed in the Materials and methods section recovered *Cestrum haberii* as most similar to *Cestrum poasanum* and *Cestrum rugulosum* Francey. The two species can be distinguished based on leaf, inflorescence and flower morphology as summarised in Tables 9 & 10.

**Table 9.** 

Characters	*Cestrum haberii*	*Cestrum poasanum*
Leaf lamina length?	55–250 mm	18–40 mm
Secondary vein no.	6–13 pairs	4–7 pairs
Inflorescence peduncle length	sessile to 2.25 mm	3–60 mm
Pedicel length	0.375–0.750 mm	0.375–2.50 mm
Flower colour (when living)	yellow, yellow–green, pale green or cream, the lobes occasionally flushed purple	white to pale green, occasionally flushed purple

**Table 10.** 

Characters	*Cestrum haberii*	*Cestrum rugulosum*
Stem and lower leaf surface hair type	branched-dendritic	simple
Petiole disposition	borne on a short woody crescent-shaped spur	not borne on a short woody crescent-shaped spur
Peduncle length	sessile to 2.25 mm	2.25-22 mm

#### Conservation status.

Using IUCN criteria (IUCN 2001) *Cestrum haberii* is considered Vulnerable (VU, A1c) under criteria A1(decline in Extent of Occurrence). A current decline in Extent of Occurrence of 27% is inferred from a projection of the locality coordinates on Google Earth (Google Earth, accessed Dec 16 2010, images 2001, 2006). Of the 15 point localities for this species, five remain as undisturbed forest, four (27%) are pasture or crop fields and eight are remnant forest patches. Collection notes indicate that *Cestrum haberii* persists in disturbed or secondary vegetation indicating a tolerance to disturbance. It is therefore assumed that this species will only be lost from a locality where vegetation cover is removed. Using this assumption the extent of decline is estimated at 27% and is assessed as ongoing. It is therefore highly likely that the decline in Extent of Occurrence will exceed 30% in the near future, meeting criteria A1c.

#### Paratypes.

COSTA RICA. Alajuela: Cantón San Ramón, La Palma de San Ramón, 10°07'12"N, 084°33'00"W (DMS), 1300 m, 30 May 1927, *A. M. Brenes 5542* (F); Cantón San Ramón, La Palma de San Ramón, 10°07'12"N, 084°33'00"W (DMS), 1100 m, 27 Jun 1927, *A. M. Brenes 5560* (F); Cantón San Ramón, Los Angeles de San Ramón, Finca Johanson, 10°12'36"N, 084°34'48"W (DMS), 1000 m, 14 Apr 1928, *A. M. Brenes 6130* (F); Cantón San Ramón, Los Angeles de San Ramón, Finca Johanson, 10°12'36"N, 084°34'48"W (DMS), 1000 m, 4 May 1928, *A. M. Brenes 6157* (F); Cantón San Ramón, Piedades Norte, Los Angeles de San Ramón, 10°12'36"N, 084°34'48"W (DMS), 1000 m, 6 Mar 1929, *A. M. Brenes 6713* (F); Cantón San Ramón, Piedades Norte, Los Angeles de San Ramón, 10°12'36"N, 084°34'48"W (DMS), 1000 m, 6 Mar 1929, *A. M. Brenes 6726* (F); Cantón San Ramón, Piedades Sur, 1100 m, 8 Mar 1933, *A. M. Brenes 17167* (F); along road between San Ramón and Balsa, at Angeles Norte, 10°08'24"N, 084°28'48"W (DMS), 1250 m, 2 Feb 1979, *T. B. Croat 46848* (MO); Monteverde Reserve, Atlantic slope, Río Peñas Blancas valley, 10°17'41"N, 084°46'50"W (DMS), 1400 m, 20 Oct 1984, *W. A. Haber 710* (MO); Reserva Biológica Monteverede Río Peñas Blancas, 10°19'N, 84°44'W (DMS), 900 m, 15 Apr 1988, *W. A. Haber & E. Bello C. 8361* (MO); Cantón San Ramón, Rancho La Paz, 10°08'51"N, 084°31'54"W (DMS), 1100 m, 15 Nov 1973, *L. J. Poveda 772* (MO); Cantón Alfaro Ruiz, La Peña de Zarcero, 1400 m, 6 May 1938, *A. Smith H488* (F); Cantón Alfaro Ruiz, La Peña de Zarcero, 1450 m, 11 May 1938, *A. Smith NY568* (F); Cantón San Carlos, Zapote, 22 Apr 1938, 1500 m, *A. Smith H649* (F); Cantón Alfaro Ruiz, El Silencio de Zarcero, 1400 m, 11 Jan 1939, *A. Smith NY1439* (F); Cordillera Central near San Juan de Laja ca. 15 km N of Zarcero, 10°15'00"N, 084°24'36"W (DMS), 1350 m, 7 Feb 1965, *L. O. Williams, A. Molina R., T. P. Williams & D. N. Gibson 29022* (F, MO); Cartago: Cantón de Cartago, Llano Grande, junto a la carretera, cerca del Río Tiribí, 09°57'00"N, 083°55'48"W (DMS), 1400 m, 17 Mar 1993, *Q. Jiménez & L. Poveda 1195* (BM, INB). Guanacaste: Cantón de Tilarán, Zona Protectora Tenorio, Cordillera Tilarán, Tierras Morenas, Río San Lorenzo, 10°36'36"N, 084°59'24"W (DMS), 1000 m, 23 Mar 1993, *G. Rodríguez & Q. Jiménez 123* (BM, INB, MO); Heredia: above San José de la Montaña on W slope of Volcán Barba, 2100 m, 17 May 1966, *F. R. Fosberg, W. H. Hatheway & D. H. Nicolson 47832* (US). Puntarenas: ca. 2 km SE of Monteverde, on the Pacific watershed, 1500–1550 m, 18–21 Mar 1973, *J. L. Gentry & W. C. Burger 2680* (F); San Vito, 08°40'N, 082°59'W (DMS), 1400 m, Jun 1974, *W. A. Haber SV#14* (MO); Monteverde Reserve, 10°18'34"N, 084°48'15"W (DMS), 1500 m, 1 Jun 1985, *W. A. Haber & E. Bello C. 1603* (MO); Cantón de Puntarenas, Monteverde, on Pacific slope, 10°18'N, 84°48'W (DMS), 1400–1500 m, 5 Mar 1992, *W. A. Haber 11036* (MO); Cantón de Golfito, Parque Nacional Corcovado, Finca Alajuela, Piedras Blancas, Sector Esquinas, 08°46'N, 083°15'W (DMS), 100 m, 19 Jun 1994, *F. Quesada 939* (BM, INB); San José: N Cordillera Talamanca, region of Cerro de la Muerte, Carretera Nacional 2, 13.5 km N of San Isidro, 09°27'36"N, 083°42'00"W (DMS), 1600 m, 4 Apr 1978, *C. Davidson 7220* (US); Santo Domingo de Vara Blanca, 2200 m, 22 Feb 1937, *M. Valerio 1552* (F). PANAMA. Chiriquí: Bajo Chorro, 08°51'N, 082°31'W (DMS), 1900–2200 m, 21 Mar 1977, *W.G. D'Arcy 10926* (MO); Boquete District, Chiquero, 08°39'N, 082°20'W (DMS), 1700 m, 11 Apr 1938, *M. E. Davidson 558* (A, F).

### Cestrum knappiae

A.K. Monro sp. nov.

urn:lsid:ipni.org:names:77116666-1

http://species-id.net/wiki/Cestrum_knappiae

Fig. 7 A–D 

#### Diagnosis.

Most similar to *Cestrum acuminatum* Francey from which it can be distinguished by the membranous to subchartaceous leaves, flowers with shorter calyces and the larger fruit.

#### Type.

**Panama.** Chiriquí/Bocas del Toro: along Continental Divide on trail in Zona Protectora Palo Seco, 08°47.1'N, 082°13'W (DMS), 1100–1300 m, 11 Aug 2000, *S. Knapp & J. Mallet 9175* (holotype: BM000648809; isotypes: MEXU, MO-913564, PMA, SCZ).

#### Description.

Shrub or small tree to 1–7 m. Leaf–bearing stems yellow–brown, pale brown to tan, the internodes 8–25(33) × 0.75–5.0 mm; young stems glabrous. Axillary buds 0.75–6.0 mm, very dark green, green–brown, orange–brown, sparsely pubescent or glabrous, not subtended by a minor leaf. Lamina 27–210 × 8–43 mm, length width ratio 2.6–8.4, narrowly lanceolate, lanceolate, narrowly ovate or narrowly oblong, membranous to subchartaceous, occasionally chartaceous, green, dull green, yellow–green; the upper surface glabrous, primary, primary and secondary veins clearly visible to the naked eye, primary and secondary raised; the lower surface glabrous; primary, primary to tertiary or primary to secondary veins clearly visible to the naked eye, primary, primary and secondary veins raised, the veinlets visible or not, secondary veins 7–20(27) pairs, 75–90° to the midrib, weakly curved; base obtuse, acute, asymmetrical, acute / obtuse, obtuse / subcordate, acute / attenuate, acute / cuneate, obtuse / decurrent; margin entire, occasionally irregularly so; apex acuminate, subcaudate to caudate; petioles regular, 3–21 × 0.675–1.0 mm, dark green, brown or yellow–green, glabrous. Inflorescences 5–9 per herbarium sheet, axillary on apical portion of the stem, terminal, pendant, 55–150 mm long, bearing 3–10 flowers in a compact panicle of 2–7 clusters of flowers, each cluster bearing 1, 2 or 4 flowers; peduncle 15–70 × 0.50–0.75 mm, straw coloured or orange–brown, glabrous; bracts 16–60 × 3–14 mm, leaf–like; bracteoles 4.0–7.5(14.0) mm, linear, spathulate, narrowly ovate, 1 or 2 per flower, glabrous. Flowers pedicellate or subsessile, where pedicellate the pedicels 0.25–1.0 mm; calyx 2.0–2.5 × 1.75–2.675 mm, the outer surface glabrous, the lobes 5, 0.125–0.675 mm, erect; corolla pale green, white or yellow–green, 28–35 mm, the tube 20–28 mm long, 2.5–3.5 mm in diameter at the mouth, 0.75–1.0 mm at the base, glabrous, the lobes 5, 6–10.0 mm long; stamens 5, the filaments 21–25 mm long, equal, adnate for 17–21 mm, with a lobe–like appendage present at insertion point, pubescent at insertion point, the anthers 0.675–1.0 × 0.5–0.75 mm; style 21–26 mm, the stigma 0.375–0.750 × 0.674–2.0 mm. Infructescences 27–110 mm long, bearing 2–4 fruit; fruiting calyx 2.0–2.5 × 3.0–4.0 mm; fruit 7–14 × 7–12 mm, white when ripe. Seeds (2)6–10, 4.5–7.5 × 1.5–5.0 × 2.5–4.5 mm, the surface smooth.

**Figure 7.**
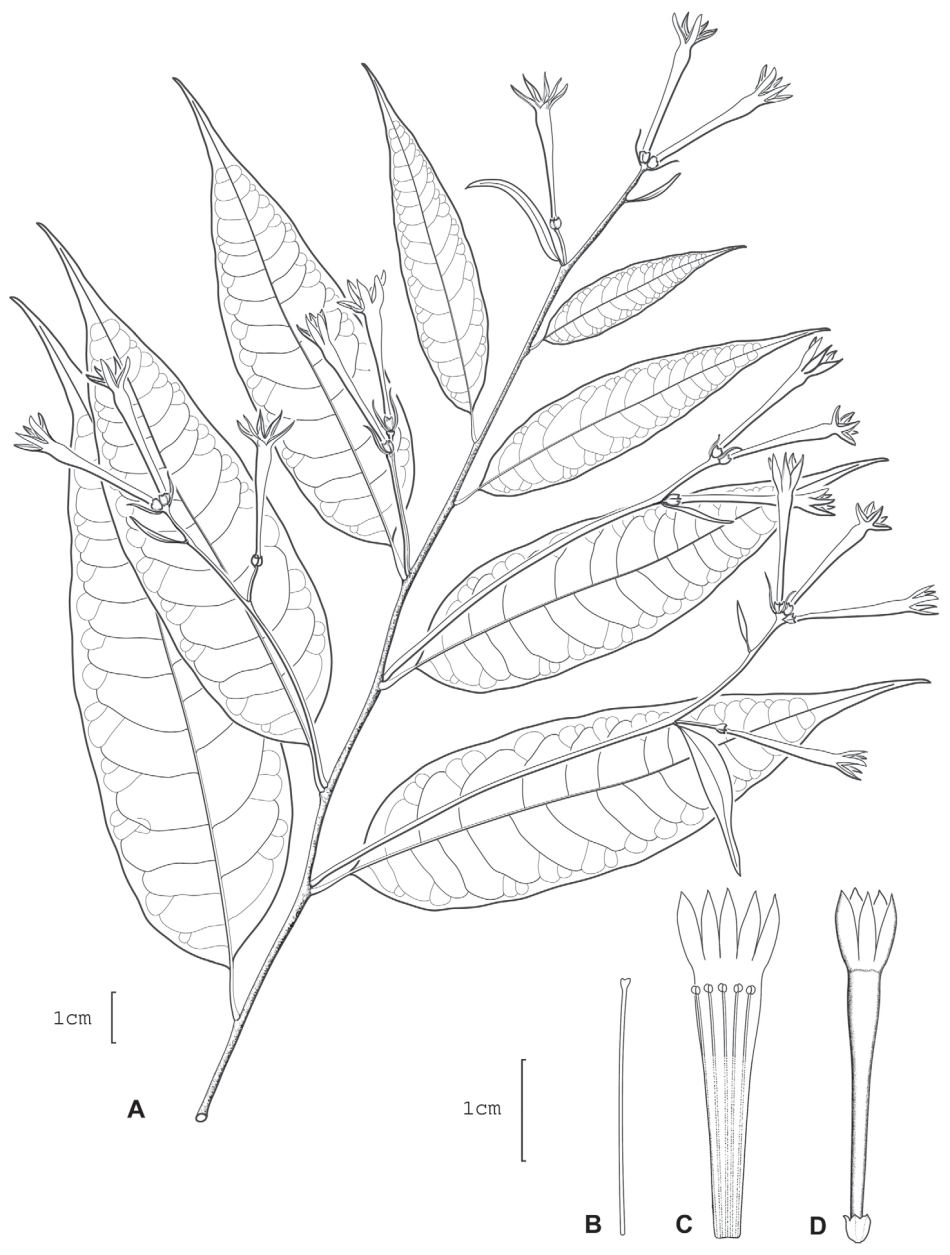
*Cestrum knappiae*A.K.Monro **A** Flowering stem **B** Style **C** Longitudinal section through the corolla showing stamen arrangement **D** Flower. Drawn from *S. Knapp & J. Mallet 9175* (BM).

#### Etymology.

This species is named after Sandra Knapp (1956–), Anglo–US botanist and Solanaceae specialist who collected the holotype and three of the paratype collections.

#### Distribution.

Tropical wet, premontane and montane forest from 1100 to 1600 m. Collection notes indicate that this species is known from primary or undisturbed forest. Existing collection localities suggest that *Cestrum knappiae* is restricted to an area of the Main Divide (river drainage between the Caribbean Sea and Atlantic Ocean) of the Talamanca Mountains ca. 270 km in extent that runs from Parque Nacional Tapantí in W in Costa Rica to the Fortuna Forest Reserve and Palo Seco Protected Areas in the E in Panama. The Extent of Occurrence is calculated to be 5,400 km^2^ (Google Earth, accesssed June 2 2011, images 2001 to 2006).

#### Discussion.

Seven of the 15 collections of *Cestrum knappiae* examined had been previously determined as *Cestrum fragile* Francey. A comparison of the holotype and paratype material with type specimens from the herbaria listed in the Materials and methods section recovered *Cestrum knappiae* as most similar to *Cestrum acuminatum* D’Arcy, *Cestrum fragile* and *Cestrum cristinae* D.A.Soto. It can be distinguished from those species based on size, leaf, flower and fruit morphology as summarised in Tables 11, 12 and 13.

**Table 11.** 

Characters	*Cestrum knappiae*	*Cestrum fragile*
Stem pubescence	glabrous	sparsely pubescent or moderatley pubescent
Minor leaves borne on truncated side branches	absent	present, occasionally absent
Secondary veins	borne at 75-90° to the midrib	borne at 45-75° to the midrib
Peduncle pubescence	glabrous	pubescent
Bracteoles	4.0-7.5(14.0) mm	1.25-4.0 mm
Infructescence fruit no.	2-4	12-36
Fruiting calyx length	2.0-2.5 mm	3.0-5.5 mm

**Table 12.** 

Characters	*Cestrum knappiae*	*Cestrum acuminatum*
Plant height	1–7 m	1–2 m
Leaf length	27–210 mm	42–107 mm
Leaf length : width ratio	2.6–8.4	2.4–3.5
Leaf texture	membranous to subchartaceous	Chartaceous to subcoriaceous
Inflorescence flower no.	3–10	4–31
Calyx length	2.0–2.5 mm	5–7 mm
Calyx lobe length	0.125–0.675 mm	1–3 mm
Fruiting calyx diameter	3.0–4.0 mm	4.5–5.5 mm
Fruit size	7–14 × 7–12 mm	5.0–6.5 × 4.5–6.0 mm

**Table 13.** 

Characters	*Cestrum knappiae*	*Cestrum cristinae*
Leaf texture	membranous to chartaceous	subchartaceous to subcoriaceous
Peduncle diameter	0.5–0.75	ca 1.5
Bracteole pubescence	glabrous	apically pubescent
Pedicel colour	pale yellow-brown, dull yellow or dull yellow-green	brown to dark brown almost black
Calyx length	2.0–2.5 mm	4.0–4.75 mm
Calyx lobe length	0.125–0.675 mm	1.25–2.25
Corolla lobe length	6–10 mm	4.75–7.0 mm
Calyx in fruit length and diameter	2.0–2.25 × 3–4 mm	4.5–6.5 × 5–7 mm

#### Conservation status.

Using IUCN criteria (IUCN 2001) *Cestrum knappiae* is considered Near Threatened (NT). *Cestrum knappiae* meets criterion B1 (Extent of Occurrence <20,000 km^2^) and one subcriterion, a (number of localities <10). The full extent of the species’ Extent of Occurrence, however, is located within protected areas. There is considerable pressure for copper and gold mining within Costa Rica and Panama within the Extent of Occurrence which may present a threat of fragmentation or decline in the near to medium term future. If mining were to take place within this area then it is likely that *Cestrum knappiae* would be classified as Vulnerable.

#### Paratypes.

COSTA RICA. Cartago: Cantón de Paraíso, Tapantí Nacional Park, Río Reventazón water basin, Tapantí station, Arboles caidos trail, 09°44'53"N, 083°46'55"W (DMS), 1600 m, 10 Jan 1997, *A. Rodríguez, S. Salas & A. Soto 1874* (BM). PANAMA. Bocas del Toro: Fortuna Dam area, Continental Divide, ridge trail to unnamed peak to E of Oleoducto road, 08°46'19"N, 082°11'51"W (DMS), 1200 m, 1 Aug 1984, *H. W. Churchill 5861* (MO); 12 miles beyond Campamento Chami (12+12 mi from Río San Félix), 08°33'N, 081°48'W (DMS), 1400–1470 m, 20 Jun 1986, *W. G. D'Arcy 16274* (MO); ca. 5 km ENE of Cerro Pate Macho near Finca Serrano, NE of Boquete, in forest along trail downhill from Finca Serrano, 08°50'24"N, 082°19'18"W (DMS), 1500 m, 12 Feb 1979, *B. Hammel 6162* (MO); on gravel road branching N from main Fortuna Dam–Chiriquí Grande road, 1.1 miles from junction, 08°47'18"N, 082°14'00"W (DMS), 1200 m, 11 Mar 1985, *G. McPherson 6801* (MO); along trail on divide separating Chiriqui and Bocas del Toro, 08°46'42"N, 082°12'48"W (DMS), 1150 m, 22 Oct 1985, *G. McPherson 7203* (MO); along trail on divide separating Chiriqui and Bocas del Toro, 08°46'42"N, 082°12'48"W (DMS), 1150 m, 22 Oct 1985, *G. McPherson 7205* (MO); N of Fortuna Dam on road to Chiriquí Grande, forested slopes along divide–trail, 08°46'36"N, 082°13'00"W (DMS), 1150 m, 18 Jan 1986, *G. McPherson 8095* (MO); vicinity of Fortuna Dam. Trail along continental divide, 08°46'12"N, 082°09'00"W (DMS), 1300–1400 m, 6 Feb 1987, *G. McPherson 10384* (MO); Bocas del Toro–Chiriquí:along Continental Divide on trail in Zona Protectora Palo Seco, 08°47'N, 082°13'W (DMS), 1100–1300 m, 11 Aug 2000, *S. Knapp & J. Mallet 9182* (BM, MO, PMA, SCZ); along Continental Divide on trail in Zona Protectora Palo Seco, 08°47'N, 082°13'W (DMS), 1100–1300 m, 11 Aug 2000, *S. Knapp & J. Mallet 9188* (BM, MO, PMA, SCZ); Chiriquí: Distrito de Guanaca, Cordillera Central, Trocha, 08°47'N, 082°14'W (DMS), ca. 1000 m, 27 Aug 1993, *M. Correa, E. Montenegro, H. Navarrete & E. Hidalgo 9836* (PMA); ridges above and W of Quebrada Aleman, trail to town of Fortuna, 08°45'N, 082°13'W (DMS), 1200–1500 m, *S. Knapp & J. Mallet 9224* (BM, PMA); Cordillera Central, 7 Dec 1996, *E. Montenegro 1595* (BM); Fortuna, Trocha Cordillera Central close to the Continental Divide, 28 Oct 1997, *E. Montenegro 1799* (INB, BM).

### Cestrum lentii

A.K.Monro sp. nov.

urn:lsid:ipni.org:names:77116668-1

http://species-id.net/wiki/Cestrum_lentii

Fig. 8 A–D 

#### Diagnosis.

Most similar to *Cestrum johnniegentrianum* D’Arcy from which it can be distinguished by the secondary nerves of the lower leaf surface which are dark relative to the leaf lamina, the longer inflorescences bearing larger bracteoles and flowers with larger calyces.

#### Type.

**Costa Rica.** Cartago: 8 km SE of Tapantí, 09°43'54"N, 083°46'43"W (DMS), 1500 m, 18 Jun 1967, *R. W. Lent 1051* (holotype: MO-3279022).

#### Description.

Unbranched shrub to 1.0–1.5 m. Leaf–bearing stems grey–tan to pale brown, the internodes 20–67 × 3.5–8.0 mm; young stems glabrous. Axillary buds 2.5–7.0 mm, brown, densely pubescent, not subtended by a minor leaf. Lamina 130–200 × 70–98 mm, length width ratio 1.7–2.4, broadly ovate, elliptic or broadly obovate, chartaceous or subcoriaceous, brown, green–grey; upper surface glabrous, primary and secondary veins clearly visible to the naked eye, primary and secondary raised; the lower surface glabrous, primary to quarternary, primary to quinternary veins clearly visible to the naked eye, primary and secondary veins raised, the veinlets visible, unbranched, secondary veins 7–10 pairs, 45–60° to the midrib, curved, weakly curved, ascending; base decurrent, cuneate–obtuse, cuneate–decurrent; margin irregularly entire, minute crenate to sinutae; apex cuspidate, subcuspidate; petioles decurrent on the stem, 17–30 × 1.5–2.5 mm, dark brown, yellow–brown, tan–cream, glabrous. Inflorescences 4–15 per herbarium sheet, axillary, panicles solitary in each axil, along the full length of leaf–bearing stems and below, 25–30 mm long, bearing 5–9 flowers borne in a panicle of 2–3 clusters of flowers with reduced branches, each flower cluster bearing 1, 3–6 flowers; peduncle 3–12 × 0.75–1.0 mm, dark brown, sparsely pubescent, the hairs 0.250–0.375 mm, branched, dendritic, eglandular; bracts absent; bracteoles 3–6 mm, linear, spathulate, sparsely pubescent. Flowers pedicellate, the pedicels 0.50–0.75 mm; calyx 4.0–4.5 × 2.0–3.5 mm, the outer surface glabrous, the lobes 5, 1.25–1.75 mm long, weakly spreading; corolla green (in bud), 15–17 mm long, the tube ca. 13.5 mm long, ca. 1.5 mm in diameter at the mouth, 0.75 mm at the base, glabrous, the lobes 5, 4 mm long; stamens 5, the filaments ca. 13 mm long, equal, adnate for 12 mm, with a lobe–like appendage present at insertion point, pubescent from insertion point to the base, the anthers ca. 1.375 × 1.0 mm; style ca. 13 mm, the stigma ca. 0.75 × 1.0 mm. Infructescences 15–25 mm long, bearing 5–8 fruit; fruiting calyx ca. 3 × 4 mm; fruit immature, colour when ripe unknown. Seeds 6, mature seeds not seen.

**Figure 8.**
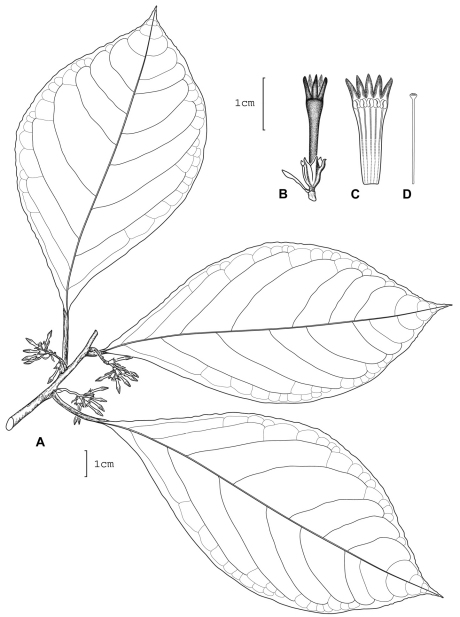
*Cestrum lentii* A.K.Monro **A** Flowering stem **B** Flower **C** Longitudinal section through the corolla showing stamen arrangement **D** Style. Drawn from *R.W. Lent 1051* (MO).

#### Etymology.

This species is named after the US collector of the holotype Roy Lent (1931–), who worked for F in Costa Rica.

#### Distribution.

Wet premontane forest from 1100 to 1700 m on Pacific and Caribbean drainage of the Talamanca Mountains, Costa Rica and Panama. *Cestrum lentii* is known from two localities ca. 210 km apart at the eastern (La Fortuna, Chiriquí, Panama) and western (Tapantí, Cartago, Costa Rica) ends of the Talamanca Mountains. It is likely that populations of *Cestrum lentii* connect these localities and that the absence of records is a reflection of sampling effort. Assuming that *Cestrum lentii* is found throughout the Talamanca Mountains between altitudes of 1100 to 1700 m then an estimated Area of Occupancy for this species is 5,880 km^2^ (Google Earth, accessed June 17, 2011, images 2001, 2003, 2006, 2009).

#### Discussion.

None of the four known collection of this species had been determined to species prior to this study. A comparison of the holotype and paratype material with type specimens from the herbaria listed in the Materials and methods section recovered *Cestrum lentii* as most similar to *Cestrum johnniegentrianum* D’Arcy. It can be easily distinguished from this species based on leaf and inflorescence morphology as summarised in Table 14.

**Table 14.** 

Characters	*Cestrum lentii*	*Cestrum johnniegentrianum*
Leaf colour when dry	brown	olive green
Lower surface secondary vein colour	darker than the lamina	equal or paler than the lamina
Inflorescence length	25–30 mm	10–21 mm
Bracteoles	3–6 mm	1.5–2.0 mm
Calyx length × diameter	3.5–3.75 × 2.0–3.5 mm	ca. 2 × 1.0–1.5 mm
Calyx lobe length	1.25–1.75 mm	0.5–0.75 mm
Filament pubescence	pubescent from the appendage to the base	glabrous
Filament appendage	present, lobe–like	absent

#### Conservation status.

Using IUCN criteria (IUCN 2001) *Cestrum lentii* is considered to beNear Threatened (NT)under IUCN (2001). The Extent of Occurrence for *Cestrum lentii* is calculated to be 5,880 km^2^ (criteria B1, <20,000km^2^, Vulnerable) and it is known from only two localities (B1a, ≤5) both of which are protected. Field experience at these localities suggests that the habitat of this species is not under any imminent threat. Illegal mining activity and planned hydroelectricity dam projects in the area may, however, result in habitat loss in the medium–term to long–term which would result in this species being assessed as Vulnerable. For this reason *Cestrum lentii* is considered Near Threatened.

#### Paratypes.

COSTA RICA. Cartago: Tapantí Hydroelectric Reserve, trail along Río dos Amigos, 09°41'24"N, 083°47'24"W (DMS), 1600–1700 m, 23 Jun 1976, *T. B. Croat 36199* (MO). PANAMA. Chiriquí: Fortuna Dam area, between Quebrada Los Chorros and Quebrada Hondo, to N of reservoir, in forest N of road, 08°45'N, 082°14'W (DMS), 1100 m, 20 Sep 1984, *H. W. Churchill & A. Churchill 6172* (MO); Fortuna Dam area, between Quebrada Los Chorros and Quebrada Hondo, to N of reservoir, in forest N of road, 08°45'N, 082°14'W (DMS), 1100 m, 20 Sep 1984, *H. W. Churchill & A. Churchill 6179* (MO).

### Cestrum talamancaense

A.K.Monro sp. nov.

urn:lsid:ipni.org:names:77116669-1

http://species-id.net/wiki/Cestrum_talamancaense

**Figure 9.**
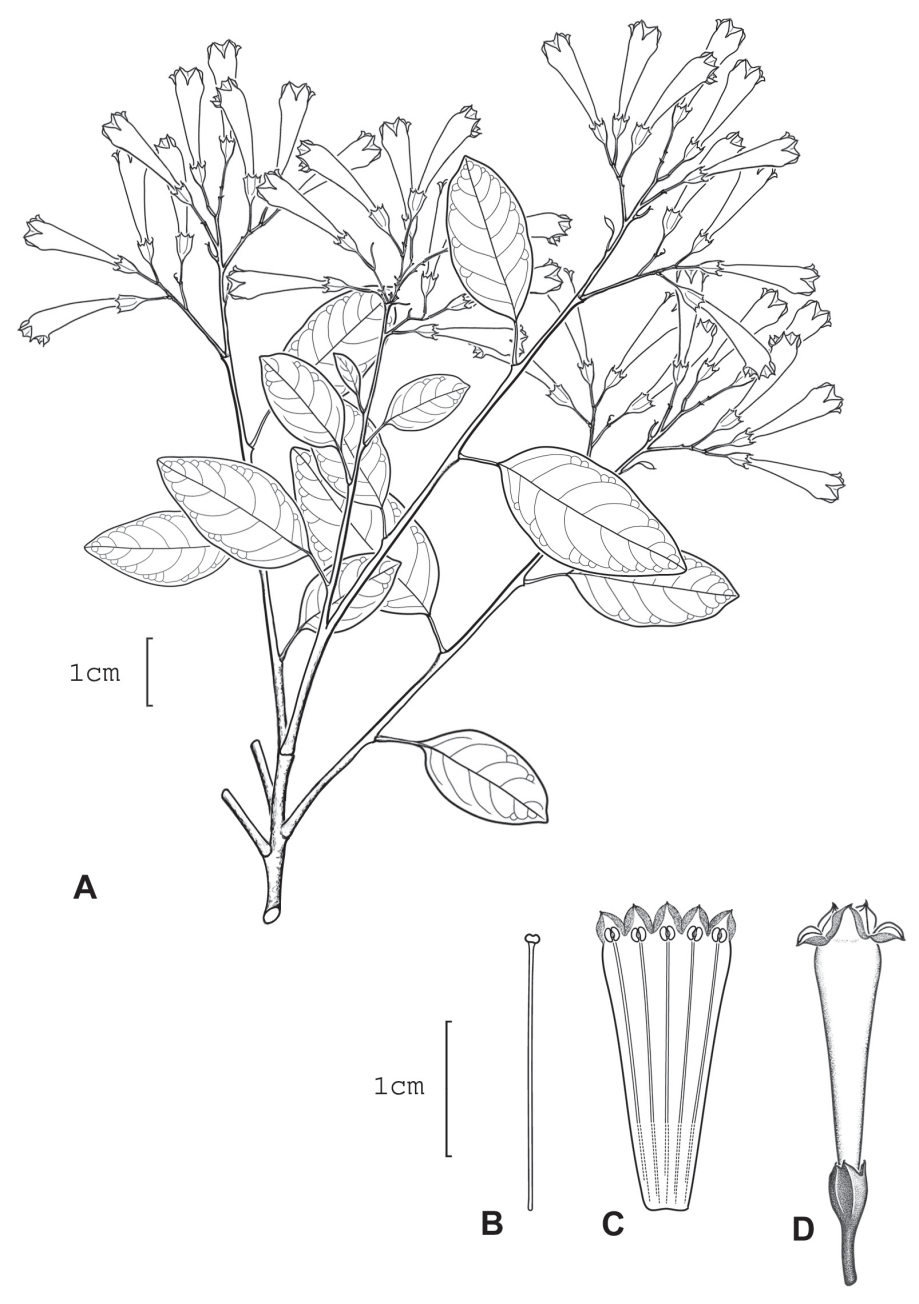
*Cestrum talamancaense*A.K.Monro **A** Flowering stem **B** Stamen **C** Longitudinal section through the corolla showing stamen arrangement **D** Flower. Drawn from *A.K. Monro, B.B. Klitgaard & J. DeGracia 4054* (BM)

**Figure 10.**
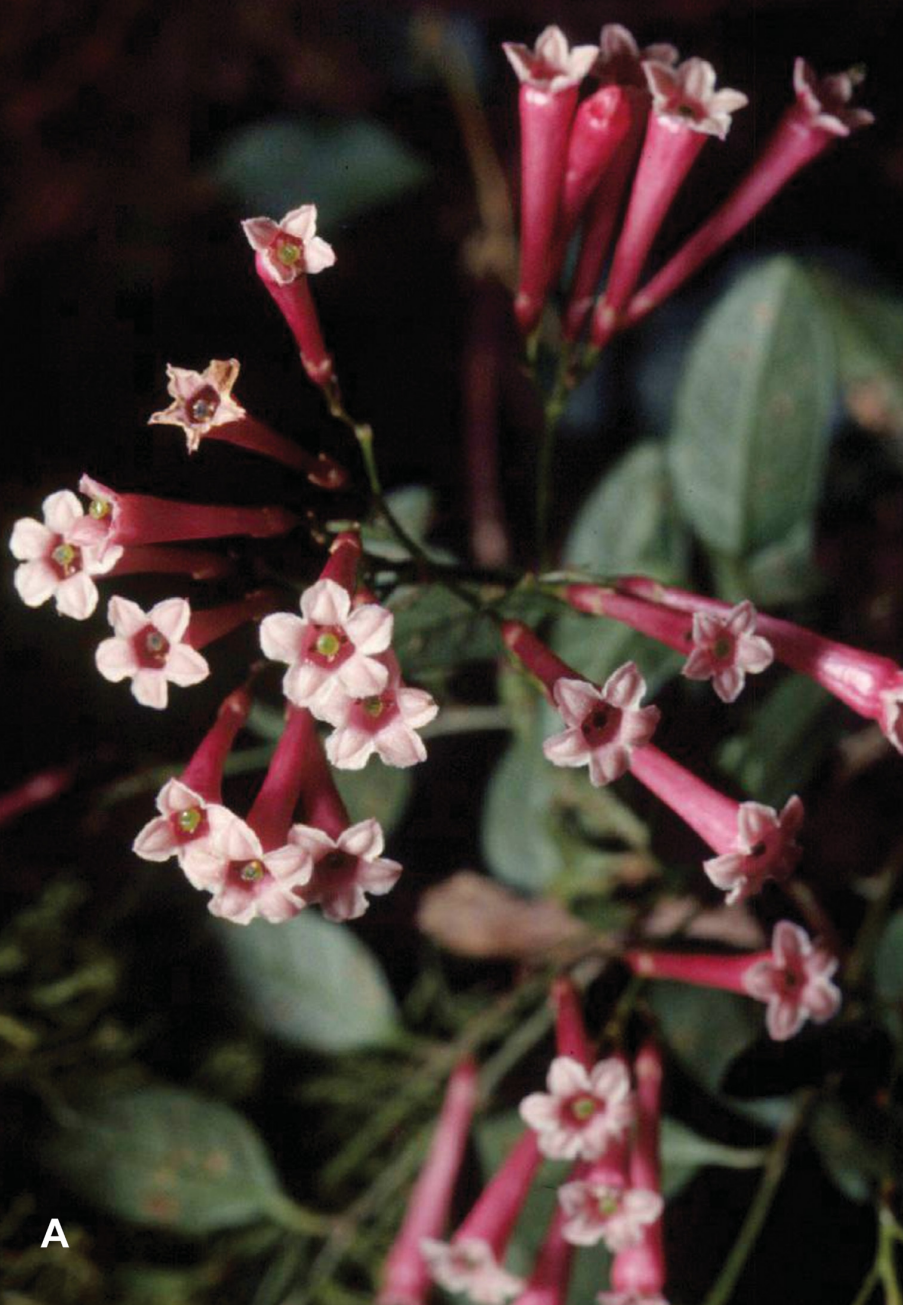
*Cestrum talamancaense* A.K.Monro **A** Inflorescence and flower. *A.K. Monro, B.B. Klitgaard & J. DeGracia 4054* (Photo A.K.Monro 2003).

Fig. 9 A–D Fig. 10 A 

#### Diagnosis.

Most similar to *Cestrum laxum* Benth. from which it can be distinguished by the absence of a minor leaf subtending axillary buds, flowers with shorter calyces that are pale pink or purple in colour.

#### Type.

**Panama.** Bocas del Toro: Cerro Fábrega, 09°04'81"N, 82°54'35"W (DDM), 3000 m, 10 Mar 2003, *A. K. Monro, B. B. Klitgaard & J. DeGracia 4054* (holotype: PMA; isotypes: BM000811836, SCZ).

#### Description.

Lax shrub to 1–2 m. Leaf–bearing stems brown, the internodes 8–20 × 1–2 mm; young stems glabrous to sparsely pubescent, the hairs 0.5 mm, simple, erect, curved, not glandular. Axillary buds 0.5–1.5 mm, black, very dark brown, sparsely pubescent, not subtended by a minor leaf. Lamina 31–80 × 15–26 mm, length width ratio 2.6–3.1, ovate, lanceolate, or obovate, chartaceous tosubchartaceous or subcoriaceous, brown; the upper surface glabrous, primary and secondary, primary veins clearly visible to the naked eye, primary and secondary, primary raised; the lower surface glabrous, sparsely pubescent, the hairs 0.25 mm, simple, erect, curved, apparently glandular, orange–brown; primary to quarternary, primary to tertiary veins clearly visible to the naked eye, primary veins raised, veinlets not visible, secondary veins 5–8 pairs, 45–60° to the midrib, weakly curved; base decurrent, asymmetrical, decurrent–cuneate; margin irregularly entire; apex acute or subcuspidate; petioles borne on a woody or crescent shaped spur, 4–13 × 0.675–1.5 mm, dark brown, glabrous. Inflorescences 6 or 7 per herbarium sheet, axillary and indeterminate terminal, solitary, clustered towards the branch tips, 40–130 mm long, bearing 6–75 flowers in a panicle of 3–12 clusters, each cluster bearing 1 or 2 flowers; peduncle 22–25 × 0.675–0.750 mm, brown, moderatley pubescent, the hairs ca. 0.5 mm, branched, dendritic, eglandular; bracts 9–19 × 2–16 mm, leaf–like; bracteoles 1.5–2.5 mm, linear, glabrous. Flowers pedicellate, the pedicels 1.25–5.0 mm; calyx 2.5–4.0 × 2.0–2.25 mm, the outer surface glabrous, the lobes 5, 3, 0.5–1.0 mm long, erect; corolla pink to pale pink, 12–23 mm long, the tube 14–18.5 mm long, 3.0–4.5 mm in diameter at the mouth, 1.0–1.5 mm at the base, glabrous, the lobes 5, 2–3 mm long; stamens 5, the filaments 7.5–16.0 mm long, equal, adnate for 4.5–8.0 mm, with a reduced swelling-like to knee-like appendage present at insertion point, pubescent from insertion point to the base, the anthers 1.0–1.25 × 0.75–1.0 mm; style 7.5–18.0 mm, the stigma 0.75 × 1.25 mm. Infructescences not seen.

#### Etymology.

From the Talamanca mountains, the locality of all known collections of this species.

#### Distribution.

*Cestrum talamancaense* is known from three locations which span the entire range of the Talamanca Mountains from Chirripó, Costa Rica in the west to La Fortuna, Panama in the east. The altitude range for this species is believed to be 2000–3200 m. This and collection label data suggest that *Cestrum talamancaense* is restricted to oak forest and subparamo vegetation. Given the small number of collections this should be considered provisional. Using this altitude range and the location of the three known localities the Area of Occupancy is calculated to be 2,300 km^2^ (Google Earth, accessed June 20, images from 2003, 2004, 2006).

#### Discussion.

Of the four collections of *Cestrum talamancaense* seen none had previously been identified to species. A comparison of the holotype and paratype material with type specimens from the herbaria listed in the Materials and methods section recovered *Cestrum talamancaense* as most similar to *Cestrum laxum* Benth. and *Cestrum irazuense* Kuntze. *Cestrum laxum* and *Cestrum irazuense* occupy a similar altitudinal range. Cestrum irazuense occupies an overlapping but broader geographical range whilst *Cestrum laxum* has a distinct and much broader geographical range being known from Mexico, Guatemala, El Salvador and Honduras. The species can be distinguished based on axillary bud, petiole and flower morphology as summarised in Tables 15 and 16.

**Table 15.** 

Characters	*Cestrum talamancaense*	*Cestrum laxum*
Axillary bud pubescence	sparsely pubescent	densely pubescent or moderatley pubescent
Axillary bud subtended by a minor leaf	not subtended by a minor leaf	subtended by a minor leaf or not
Petiole pubescence	glabrous	moderatley pubescent, occasionally glabrous
Calyx length	2.5–4.0 mm	4–15 mm
Corolla lobe length	2–3 mm	3–5 mm
Corolla colour when fresh	pale pink	yellow, pale green to yellow–green

**Table 16.** 

Character	*Cestrum talamancaense*	*Cestrum irazuense*
Stem and lower leaf surface pubescence	glabrous or sparsely pubescent	glabrous
Peduncle length	22–25	2–12
Peduncle pubescence	pubescent	glabrous
Calyx diameter	2–2.5 mm	0.75–1.75 mm
Flower colour	pink to pale pink, or purple	pale green, pale to dark purple
Corolla tube base width	1–1.5 mm	0.5–1 mm
Corolla tube mouth width	3-4.5 mm	2–3.5 mm

#### Conservation status.

Using IUCN criteria (IUCN 2001) *Cestrum talamancaense* is considered Least Concern. *Cestrum talamancaense* has an Area of Occupancy of 2,300 km^2^ (Criteria B2, >2000 km^2^) and meets a single subcriteria for criteria Ba (number of localities less than 5). In addition the whole of the range of this species is currently not fragmented and within protected areas both in Costa Rica and Panama. Should current attempts at illicit mining within the Area of Occupancy persist, however, the threat status may need to be revised to Near Threatened.

#### Paratypes.

COSTA RICA. San José: Parque Nacional Chirripó, Cuenca Térraba–Sierpe, Sabana, Chirripó, 09°25'12"N, 083°30'36"W, 3200 m, 6 Nov 1996, *E. Alfaro, B. Gamboa & A. Picado* 895 (BM, INB). PANAMA. Bocas del Toro: Parque Nacional La Amistad, Cerro Fabrega, 09°06'56"N, 82°52'15"W (DDM), 3200 m, 16 Mar 2003, *B. B. Klitgaard, A. K. Monro & J. E. DeGracia* 818 (BM, INB, MO, PMA, SCZ).); Chiriquí: Proyecto Fortuna, Valle de Hornito, 08°38'N, 082°13'W (DMS), ca. 2000 m, 13 Feb 1981, *J. J. Him & I. O. Gordon* 50 (PMA).

## Supplementary Material

XML Treatment for Cestrum amistadense

XML Treatment for Cestrum contrerasianum

XML Treatment for Cestrum darienense

XML Treatment for Cestrum gilliae

XML Treatment for Cestrum haberii

XML Treatment for Cestrum knappiae

XML Treatment for Cestrum lentii

XML Treatment for Cestrum talamancaense

## References

[B1] Benítez C D’Arcy WG (1998) The genera *Cestrum* and *Sessea* (Solanaceae: Cestreae) in Venezuela. Annals of the Missouri Botanical Garden 85 (2): 273-351 doi: 10.2307/2992010

[B2] D’Arcy WG (1973) Solanaceae. In: Woodson RE, Schery RW (Eds) Flora of Panama Family 170 Annals of the Missouri Botanical Garden 60: 573–780.

[B3] D’Arcy WG (2001) Solanaceae. In: Stevens WD Ulloa U C, Pool A, Montiel OM (Eds) Flora de Nicaragua.Monographs in Systematic Botany from the Missouri Botanical Garden 85: 2376-2424

[B4] Dunal MF (1852) Solanaceae. In: deCandolle ALLP (Ed) Prodromus 13 (1): 1-690

[B5] Francey P (1935) Monographie du genre *Cestrum* L. Candollea 6: 46-398

[B6] Francey P (1936) Monographie du genre *Cestrum* L. partie II. Candollea 7: 1-132

[B7] Google Earth 6.0.3.2197 (2011) Available from http://www.google.com/earth

[B8] Gentry JL Standley PC (1974) Solanaceae. In: Gentry JL, PC Standley (Eds) Flora of Guatemala, Fieldiana Botany 24 (10): 1-151

[B9] International Union for Conservation of Nature and Natural Resources Species Survival Commission (2001) IUCN Red List Categories: Version 3.1. International Union for Conservation of Nature and Natural Resources, Gland and Cambridge, 70 pp.

[B10] Linnaeus C (1753)*Cestrum.* In: Species Plantarum 1: 191.

[B11] Mauchamp A (1997) Threats from Alien Plant Species in the Galápagos Islands. Conservation Biology 11: 260-263 doi: 10.1046/j.1523–1739.1997.95356.x

[B12] Nee M (1986) Solanaceae. In: A Gómez–Pompa (Ed) Flora de Veracruz 49. Instituto Nacional de Investigaciones sobre Recursos Bióticos, Xalapa, 1– 191.

[B13] Nee M (2001) An overview of Cestrum. In: Van den Berg RG Barendse GWM Van der Weerden GM Marinni C (Eds). Solanaceae V: advances in taxonomy and utilization.Nijmegen University Press, Nijmegen: 109-136

[B14] Pattison RR Goldstein G Ares A (1998) Growth, biomass allocation and photosynthesis of invasive and native Hawaiian rainforest species. Oecologia 117(4): 449–459. http://www.springerlink.com/openurl.asp?genre=article&id=doi:10.1007/s004420050680 10.1007/s004420050680 28307669

[B15] Schulz OE (1909) Solanacearum genera nonulla. In: Urban I (Ed). Symbolae Antillanae 6.Bontraeger Brothers, Leipzig: 140-279

[B16] Sheppard SRJ Cizek P (2009) The ethics of Google Earth: Crossing thresholds from spatial data to landscape visualisation. Journal of Environmental Management 90: 2102-2117 doi: 10.1016/j.jenvman.2007.09.012 18599184 10.1016/j.jenvman.2007.09.012

[B17] Space JC Waterhouse BM Miles JE Tiobech T Rengulbai K (2003) Report to the Republic of Palau on invasive plant species of environmental concern. USDA Forest Service Pacific Southwest Research Station Institute of Pacific Islands, Honolulu.

[B18] Standley PC Morton CV (1938) Cestrum. In: Standley PC (Ed). Flora of Costa Rica. Fieldiana.Botany 18: 1045-1053

[B19] Sykorova E Lim KY Chase MW Knapp S Leitch IJ Leitch AR Fajkus J (2003) The absence of *Arabidopsis*–type telomeres in *Cestrum* and closely related genera *Vestia* and *Sessea* (Solanaceae): first evidence from eudicots. The Plant Journal 34: 283-291 doi: 10.1046/j.1365–313X.2003.01731.x 12713535 10.1046/j.1365-313x.2003.01731.x

